# Use of Personal Resources May Influence the Rate of Biological Aging Depending on Individual Typology

**DOI:** 10.3390/ejihpe12120126

**Published:** 2022-12-02

**Authors:** Tatiana N. Berezina, Stanislav A. Rybtsov

**Affiliations:** 1 Department of Scientific Basis of Extreme Psychology, Moscow State University of Psychology and Education, 127051 Moscow, Russia; 2 Center for Regenerative Medicine, University of Edinburgh, Edinburgh EH8 9YL, UK

**Keywords:** biological age, relative biological aging, personal resources, individuality, types of individuality, personal typology

## Abstract

Individual hobbies and interests, the ways of spending leisure time develop personal resources influencing health and wellbeing. The literature analysis helped selecting thirteen personal resources that also affect the rate of aging: sports, order, creativity, intellect, handwork, kindness, Humor, spirituality, risk, nature, achievements, optimism, communication. In 1632 people, (840 women and 792 men) personal resources were assessed using a questionnaire developed in-house. Biological age was determined by health indicators. The personal typology was determined by testing functional asymmetry, physique, interaction style, emotionality, profession, marital status, gender, age, and place of residence. The data were processed by correlation and cluster analysis and methods of automatic artificial neural networks (ANN). Personal resources were used as input continuous variables. Personality types were used as input categorical variables. The index of relative biological aging (RBA) was applied as an output continuous variable. We also calculated the correlation between the RBA index and the applied personal resources in different types of personalities. For most female types including investigative occupations, psychomotor emotionality, living in urban areas, asthenic physique, negative correlations were found between most personal resources and the aging index. In men, resources that slow down aging are found only for certain types: enterprising and conventional professions, ambidexter and left-handed, intellectual emotionality, athletic physique. In conclusion, with the help of the trained ANN, we selected personal resources that slow down aging. For women of all types, there are common resources reducing RBA index including nature, intellect, and achievements. For men, ANN was unable to find common resources that slow down aging. However, with an individual selection of resources, a trained neural network gives a favorable forecast of the ability to slow down the biological aging of a particular man by changing his hobbies and interests and ways of spending free time.

## 1. Introduction

Increasing life expectancy has stimulated retirement reforms in many countries. For a sensible increase in the retirement age, as well as for the formation of an active longevity strategy, it is necessary to identify which personal resources slow down biological aging. Researchers have identified many personal factors that affect life expectancy, health, and the rate of biological aging (intellect, optimism, having a family, children, education, a successful career, etc.). However, there are conflicting data on many factors. These factors turn out to be positive for some groups of people and indifferent or negative for others. Therefore, it is important to select psychological factors for a particular person, considering their individual typological characteristics [1].

The novelty of our study is the individual typological approach to anti-aging. We studied the correlation between personal resources and biological age for different types of people depending on gender, age, place of residence, profession, physique, emotionality, etc. Additionally, new is our definition of personal resources as activities that are additional to a person’s daily life (for example, hobbies) which are easily amenable to personal control and training.

Quantitative indicators of the rate of aging are valuable tools for assessing “healthy aging”, predicting senile diseases and human life expectancy [1]. To assess the rate of aging, phenotypic biomarkers of aging are used, such as standing balance, grip strength, walking speed, body mass index, waist circumference, and muscle mass [2,3]. Other panels of biomarkers for healthy aging (e.g., UK panel) additionally include measures of physical performance and cognitive function [4].

Biological age is determined by an integral set of indicators determined by aging biomarkers, including biochemical ones. For example, by the activity of sirtuin enzymes (SIRT), which regulate many cellular functions, including DNA repair. The presence of negative correlations between the activity of sirtuin and the rate of aging has been shown. Thus, an increase in sirtuin activity may indicate a slowdown in aging [5]. Chromosomal aging can be determined by examining telomere length and telomerase activity. Factors such as climate, nutrition, lifestyle, stress, and infections can attenuate telomerase activity and consequently reduce the rate of telomere repair [6].

A study by C. Werner showed that the inclusion of endurance training in the regular daily routine affects telomerase activity and telomere length in healthy people [7]. 

A significant advance in the analysis of biological age was the discovery of epigenetic clocks—methods for analyzing modifications of DNA regions (methylation of CpG islands) [8,9]. In another integrated model, along with the analysis of DNA methylation, a number of cellular stress factors, inflammation, and markers of senescent cells were included. This model made it possible to predict biological age, the risk of type 2 diabetes and other senile diseases. The model also showed a strong relationship with life expectancy and lifestyle, including adherence to a healthy diet and level of education [10]. Belsky laboratory based on DNA methylation and change-over-time in 18 biomarkers tracking organ-system integrity also developed a biological age determination model and compared it with other methods, including the frailty index [11]. Thus, DNA methylation analysis and the frailty index are among the best predictors of biological age and lifespan, while telomere length analysis is less predictive [12]. However, the analysis of biological age by the DNA methylation pattern remains a complex and resource-intensive analysis.

To assess biological age in biopsychological research, the frailty index is increasingly used as an integral indicator of health [13].

The frailty index usually includes an assessment of about five key health indicators: slowness, weakness, physical performance, exhaustion, weight loss, as well as indicators of the cardiovascular and respiratory systems [14]. The Institute of Gerontology of the Academy of Medical Sciences (USSR) conducted large-scale population studies of indicators of biological age. As a result, key indicators including in the Voitenko panel were developed to assess biological age. According to Voitenko, biological age is determined by the state of the cardiovascular system, respiratory system, balance organs, body weight and subjective health assessment [15].

Indicators of biological age are recognized as the most important tools for assessing “healthy aging” and for evaluating the impact of various personal resources on anti-aging [1]. Human behavior, lifestyle, daily routine, and attitude affect the biological age, determined both by bioindicators of aging and at the chromosomal level [16,17]. Indicators of biopsychological age are used to evaluate the effectiveness of preventive therapy, including physical exercises, healthy diet and individual psychotherapy, to correct a number of personal factors (e.g., optimism, communication, wildlife, etc.) [18].

All personal factors influencing indicators of aging, biological age, or health in old age can be divided into two groups: socio-demographic factors and personal resources.

For the assessment of aging, the importance of both biological and socio-demographic factors has already been recognized and emphasized in many studies [19]. Gerontologists refer to the social factors of aging as low socioeconomic status, low level of education, adversity in childhood, a lonely lifestyle, belonging to minorities, bad habits, and adverse psychological conditions [20]. Additionally, important socio-demographic factors are gender [15], age [21], type of occupation [22], unemployment [23], achievements and social status [24], marital status, presence of children and close relatives [25]. All these factors affect the rate of natural (chronological) aging of a person and the state of their health, therefore indicators of biological age are used as the best predictor [26].

We designate socio-demographic factors in our study as the individual typological characteristics of a person. They form the basis of human daily life, in fact, they are life itself. Undoubtedly social factors are very important. However, their application for targeted aging management is very difficult, since the individual typological characteristics of a person are hard to change, and these changes require a restructuring of the entire life path [27]. We suggest that, in addition to socio-demographic factors, aging management should also take into account personal resources that a person can change. We consider personal resources as compensatory configurations that a person can master and use subjectively, in addition to their daily life. We called such resources personal anti-aging resources. We give the following definition: personal anti-aging resources are features of the psyche or behavior that improve health, contribute to a slowdown in biological aging and an increase in life expectancy. Most often, they include hobbies [21]. We propose criteria for classifying an indicator as a personal resource. Firstly, these resources are subject to the control of the individual and can be arbitrarily developed and trained. Secondly, these resources are auxiliary, supplementing a person’s life path; their application does not require a radical restructuring of the whole life. Thirdly, there is empirical evidence of their effectiveness for various groups of people. As for internal and socially defined resources, we propose to consider them as individual typological personality features.

Based on the published data, we have identified 13 resources that meet these criteria. Sports (Sportive and physical activities). Most authors believe that physical activity is closely related to the biological age; a decrease in the level of physical activity (measured by the average number of steps per minute) allows predicting the acceleration of biological aging [28]. Physical activity is also associated with the immunological age [29]. Regular exercise has been shown to be correlated with reduced influenza frequency and mortality in the elderly [30,31].

Order (consciousness, life control, time management). Consciousness and the organization of the life path have an impact on human health and aging [32]. Good self-controlled time management increases the likelihood of a long life and reduces the risk of unexpected death. A well-known experiment by E. Langer and J. Rodin studied the relationship between life control and its duration. The experiment involved residents of a nursing home. Some of the older people controlled the environment, made decisions for themselves, for others, all this was done by the attendants. The Longitudinal study showed that life expectancy was longer in those older people who managed themselves [33].

Creativity (productive and reproductive creative activities). The presence of creative hobbies in middle-aged and elderly people reduces the number of depression symptoms [34], improves quality of life, general health, social functioning and other social and demographic indicators [35].

Intellect (intellectual activities, education and self-education). Evidence of the effectiveness of this resource has been obtained by many researchers. They believe that getting an education contributes to an increase in life expectancy [36]. Intellect indicators contribute to a longer, more productive life and reduce the risk of Alzheimer’s disease, even those residing in a monastery, where all other living conditions are the same [37].

Subject resource (any handwork hobby, such as needlework, etc.). In Japan, a study has shown that any hobby in both middle- and old-age is associated with a lower risk of disabling dementia without a history of stroke [21], with many elderly women doing handwork as a hobby.

Kindness (caring for others, altruism, helping and assisting activities). There are studies confirming the relationship of kindness with life expectancy. The most informative is the study by M. Poulin and colleagues; they monitored 846 subjects for 5 years. The subjects reported how much time they spent helping neighbors, friends, and/or relatives. Those who actively helped others during the previous year were less likely to die than those who did not. The authors explain that altruistic behavior creates a buffer in the human psyche, accumulating energy and thereby protecting it from the action of negative factors, and resulting in a decrease in the probability of death [38].

An analysis of five population-based studies by D Roth et al. concluded that kindness to people is associated with increased life expectancy. Despite the individual characteristics of personal typology, a decrease in mortality and an increase in life expectancy was found in people who cared for the elderly compared to a control group that did not provide such care [39].

Humor (any appeal to the humorous). It has been shown that a sense of humor can influence the duration of a productive life. S. Svebak showed on an example of 66,140 residents of the Noor-Trendelag district (Norway) that a sense of humor positively correlates with subjective health and reduces the likelihood of cardiovascular diseases, cancer, and diabetes [40]. There is also evidence of a positive effect of a sense of humor on life expectancy [41].

Spirituality (self-improvement, spiritual and religious practices). This includes both turning to traditional religions, and engaging in various practices of self-improvement, hobbies aimed at finding the meaning of life, etc. It has been proven that religiosity and observance of religious rites have a positive effect on subjective health and slow down the biological aging of representatives of dangerous professions [42].

Risk (dangerous and risky hobbies). This is an ambiguous factor. It can increase an individual’s life expectancy as well as reduce it [43]. A small risk causes positive emotions and represents environmental eustress that is a positive health factor. Studies have shown that eustress improves recovery after myocardial infarction by increasing the survival of cardiac macrophages [44].

Communication resource. Most researchers believe that communication is beneficial for health and longevity [45]. American scientists noted that life expectancy was positively related to the frequency of socialization with neighbors, and of religious participation [46]. Additionally, traditionally a favorable factor that increases life expectancy is the presence of family relationships in old age [25].

Nature. A recent review by Caoimhe Twohig-Bennett, which included a meta-analysis of 103 observational and 40 intervention studies, found that exposure to green spaces correlated with multiple health benefits [47]. It has also been shown that there is a significant moderate link between the spatial distribution of green spaces in cities and the risk of mortality. However, the negative association between interconnected landscape areas and all-cause mortality varies by age and education, the association being stronger for areas with a higher percentage of older and less-educated adults [48].

Achievements (activity resource of success and victories). Many researchers consider success in life to be a personal resource that affects longevity. British psychologists have studied employees of the British Civil Service and found that the highest life expectancy was among top-level administrators, and the lowest was among ordinary workers [24]. The cosmonauts who have travelled into Space lived longer than those who were in the reserve and did not fly into Space [49].

Optimism. Most authors suggest an optimistic attitude to life a factor of longevity. According to Mayo Clinic researchers, optimists have a 19% longer life expectancy [50].

Thus, the effectiveness of indicated resources has been empirically proven for certain groups of people clustered by age, gender, and place of residence, as well as for the certain types of personality.

Researchers note that the action of antiaging factors is not always unambiguous and depends on many conditions: age [15], gender [21], personality characteristics [51], type of profession (physical or intellectual work) [52], the presence or absence of work [23], and place of residence (living in the central or peripheral region of the country) [43].

H. Wang & L. Tassinary believe that the effectiveness of the nature resource depends on age and education [48]. Matsumura et al. showed that the resource of having a hobby reduces the risk of dementia depending on the age group and the presence of cardiovascular diseases [21].

We suggest that these indicators (age, gender, place of residence, presence of a family, children, etc.) be considered as individual typological personality traits through which resources affect biological aging.

## 2. Materials and Methods

The purpose of our research was to study the correlation of personal resources with the index of relative biological aging (RBA), depending on the individual typological characteristics of the respondent.

### 2.1. Participants

The study involved 1632 people, including 840 women and 792 men. All subjects were of pre-retirement age: women were from 36 to 55 years old; men were from 36 to 60 years old.

To obtain a generalized sample, residents of different regions of Russia were surveyed: Moscow and Moscow region, Orenburg region, North Ossetia, Ufa and the Republic of Bashkortostan. The subjects were selected on a territorial basis. One residential area was selected in the surveyed city or village (a site assigned to a medical or educational institution). If the plot was large, then a part of it (a block of flats, a street) was randomly selected. All people living in this area and meeting the criteria for the selection of subjects were invited to participate in the study. There were four criteria for selecting subjects. The first criterion was age. Only people of the studied age group (36–55 women and 36–60 men) were invited for the investigation. The second criterion was consent. Only people who gave voluntary and informed consent took part in the study. The third criterion was anonymity. The study was conducted anonymously. Participants’ personal data was deleted after data collection. The fourth criterion was subjective confidentiality. The subjects could not report some information if they considered it confidential (most often, the subjects did not want to disclose age, weight, body type, professional type, etc.). Confidential data was not entered into the matrix and was not considered when processing the data.

### 2.2. Instruments

1. Biological age assessment. The author of the technique is V.P. Voitenko [53,54]. For the calculation, objective physiological parameters of the body are used, including arterial systolic pressure, arterial diastolic pressure, duration of breath-holding after a deep breath (in men), body weight (for women), and static balancing on the left leg (without training), as well as subjective indicators, such as (subjective) self-assessment of health (28 questions about the presence of psychosomatic symptoms). The expected biological age (EBA) (the statistical norm for a given age), as well as the rate of aging of the organism (the difference between biological age and expected biological age) are also calculated.

For further analysis, we used only the last indicator: the index of relative biological aging (RBA), which is the difference between biological age and expected biological age (the method is described in detail here [54]).

The difference between biological age and expected biological age in the range from −15 to −5 characterizes a slower rate of aging; within the range from −4.99 to +4.99 is the natural rate of aging; the difference between +5 to +15 is premature aging. The validity of the method has been verified in a series of studies in Russia [22], as well as in cross-cultural studies [54]. The authors assessed the structural reliability of the biological age (method by Voitenko). They calculated the correlation coefficient between the variables that are part of the methodology. They showed a good correlation between all the indicators, which shows the structural reliability of the test [42]. Before we started our study, we assessed the retest reliability of the biological age (BA) measurement method using the Pearson’s correlation coefficient. It was between 0.6–0.7. A detailed description of the retest reliability is reported here [42].

2. Test questionnaire of personal resources was developed specifically for this study. It measured the respondent’s use of various personal resources over the last year. The questionnaire had 13 scales and contained 65 questions. Questionnaire scales for resources: (1) Sports, (2) Order, (3) Creativity, (4) Intellect, (5) Subject resource (Handwork), (6) Kindness (Altruistic resource), (7) Humor, (8) Spirituality, (9) Risk, (10) Communication, (11) Nature, (12) Achievements, (13) Optimism.

This method was preliminarily standardized on another sample. The results of standardization and the method itself are presented here [55]. The structural reliability of the questionnaire was confirmed by the calculation of Cronbach’s alpha (0.4–0.6). Retest reliability was in the range of 0.7–0.8. Criteria validity was in the range of 0.2–0.5.

3. Personality types were identified using the following methods.

- Questionnaire (gender, age, having family, children, place of residence, and profession).

- The preferred interaction/communication style (cooperation, competition, or compromise) was determined using a game based on the prisoner’s dilemma by Rapoport [56]. The subjects were offered four situations in which they could choose either a cooperating or competing way of solving the dilemma. A person who chose rivalry 3–4 times was referred to the competitive style. Those who chose collaboration four times were assigned to their preferred style: collaboration. In other cases, the participant was categorized as the intended style: compromise the game is presented online in Russian: http://95.181.226.63/ (accessed on 28 November 2022).

- Body type (ectomorph (asthenic), endomorph (picnic), mesomorph (athletic), indeterminate (harmonious). This was determined based on functional measurements of weight, height, wrist volume, shoulder width, hips. An indefinite type was set if there were difficulties with assigning a shape to one of the types.

- Type of functional asymmetry (right-handed, left-handed, or ambidexter) determined by the dominant hand. The subject was asked to conduct a series of tests (applause, crossing the arms on the chest, interlacing fingers, in which hand the subject holds a phone) and report the result to the psychologist. If the right hand dominated in most samples, the right-handed type was set. If the left hand dominated, the left-handed type was set. If dominance changed, then the ambidexter type was set.

- Type of emotionality according to V. M. Rusalov (communicative, intellectual, and psychomotor) was determined using the structure of temperament questionnaire (STQ) test [57].

### 2.3. Procedure

The research program was approved by the Ethical Commission of Psychological and Interdisciplinary Research of the Faculty of Extreme Psychology of the Moscow State Psychological and Pedagogical University (Moscow, Russia), protocol No. 59k-03/19 of 23 April 2019 and was compiled in accordance with the rules of the 1975 Declaration of Helsinki (as amended in 2013) and was also guided by the ethical code of psychologists when conducting psychological research.

All potential subjects (adults living in the study area) were contacted by psychologists orally, by phone or by e-mail. The examination of biological age was carried out individually with each participant. Diagnosis of personality types was carried out using Google questionnaires. Based on the responses from the subjects, the psychologists determined their personality types. All studies were conducted anonymously. Participants gave written voluntary informed consent to participate in the study.

### 2.4. Analysis

From the collected data, separate tables were compiled for each type, separately for men and women. The tables included the RBA index and indicators of personal resources. Tables were assembled for the following types (See Supplementary Tables S1–S16). By the presence of a family (single, divorced, married). By the presence of children (childless, having children). By professional type (realistic, investigative, social, conventional, enterprising, or artistic type). By place of residence (living in rural or urban areas, or in the capital). By physique (asthenic, picnic, athletic or indefinite (harmonious) types). By type of relationship with other people (competitive, compromising, or collaborating). By emotionality (psychomotor, intellectual, or communicative). By hand asymmetry (right-handers, ambidexters, or left-handers).

Further, for each type, the Spearman’s correlation coefficients between the indicators of biopsychological aging and personal resources were calculated. If there were no respondents of some type, or there were few of them, the correlation coefficients were not calculated (See Supplementary Tables S1–S16).

The obtained Spearman’s correlation values between RBA index and personal resources by different typology were clustered using the Pearson’s correlation method and visualized using CIMminer (web based software: https://discover.nci.nih.gov/cimminer/oneMatrix.do (accessed on 28 November 2022).

For a general assessment of the impact of personal resources on the RBA index of biological aging, we used multiple regression with stepwise exclusion. Two regression equations were calculated separately for men and women. The dependent variable was the RBA index, and the independent variables were personal resources.

To generalize the results and create a simulation model, we used the method of automatic artificial neural networks (ANN) from the statistical software package Statistica 12 (StatSoft). The “regression” method was used to develop the neural network. Since most age-related indicators do not have a normal distribution, we chose a statistical method that was independent of distribution—the method of automatic neural networks. The following parameters were set for creating and training the neural network: network type MPP (massively parallel processing). The minimum number of hidden neurons (1). The maximum number of hidden neurons (1). Networks for learning (20). Networks for conservation (5). Finally, one network was selected showing the maximum correspondence to the initial empirical data in the test study. Subsample size (random). The training network was built on 70% of the sample, the control network on 15%, and the test network on 15% of the sample.

Continuous input variables—personal resources: (1) Sports, (2) Order, (3) Creativity, (4) Intellect, (5) Handwork (Subject resource), (6) Kindness (Altruistic resource), (7) Humor, (8) Spirituality, (9) Risk, (10) Communication, (11) Nature, (12) Achievements, (13) Optimism.

Categorical input variables—the personality types described above.

The output continuous target variable (analogous to the dependent variable for regression analysis) was the index of biological aging (RBA—difference between biological age and expected biological age). The methodology for calculating the RBA index is described in Section 2.2.

As a result, the trained neural network was calculated. In women, it was a network with a learning algorithm: BFGS 61 (Broyden–Fletcher–Goldfarb–Shanno), with the activation function of hidden neurons (hyperbolic) and with the activation function of output neurons (exponential). For men, it was a network with a learning algorithm: BFGS 80, with the activation function of hidden neurons (hyperbolic) and the activation function of output neurons (identical).

The capabilities of the neural network for the selection of personal resources were evaluated considering the personal types of the subjects. At the first stage, the neural network evaluated the contribution of all variables to the aging of the individual (determining the weight of the variable). Thus, a series of weights for each personal resource and individual type was calculated. We arranged all variables in descending order of their favorableness: from the most negative value (anti-aging) to the most positive (accelerating aging).

At the second stage, the simulation of the use of various resources by an individual was carried out. The simulation was carried out for 100 randomly selected men and 100 women. It was studied how the biological aging index changed in the following situations. (1) If all subjects are given recommendations for a general increase in the use of “favorable” (slowing biological aging) resources by 1 point and they will follow the recommendations. (2) If subjects are given recommendations on the overall reduction of “unfavorable” (aging-accelerating) resources by 1 point and they will follow the recommendations, (3) If for each subject an individual selection of favorable resources is carried out, guided by the previously calculated correlation values and taking into account all their typological features. These favorable resources (which had negative correlations with aging) were increased by 1 point and unfavorable (adverse) resources were decrease by 1 point for all individual types characteristic of a particular subject. Note that the increase or decrease was conducted for each individual subject according to their typology, and not for all subjects, as in the previous situations. The trained ANN was saved for future use.

## 3. Results

### 3.1. Multiparametric Analysis of Correlations of Various Personal Typologies

**Figure 1:**
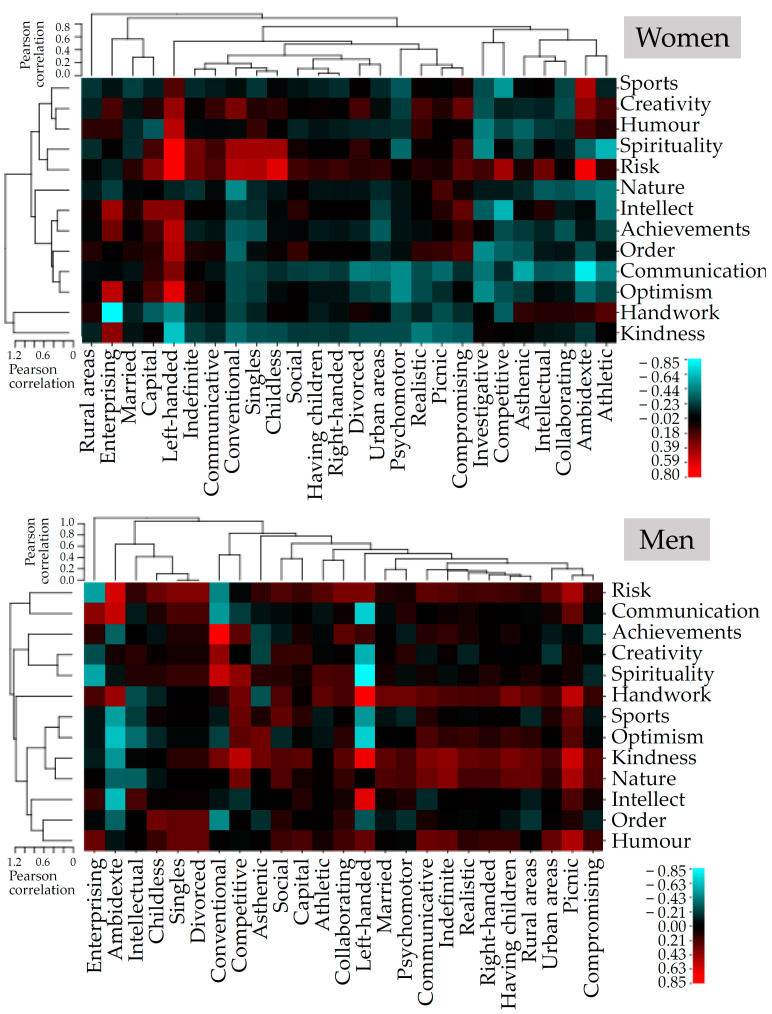
Cluster analysis correlation of RBA index with scales of personal resources and typologies.

Correlations between RBA index and the scales of personal resources for different typology groups was generalized by cluster analysis using Pearson’s correlation method. Clustered values were presented as a heat map separately for men and women. As mentioned above, the Risk resource is positively correlated with aging to a greater extent for women and to a lesser extent for men (Figure 1, red rows). The Risk resource affects the aging of almost all typological groups in women and is neutral only for those living in rural areas and for the enterprising type of profession. Moreover, for a number of female typologies (living in capitals, left-handed, indefinite body type, communicative, conventional occupation, single or child-less) form a cluster associated with accelerated aging. This cluster was also associated with resources, spirituality, humor and creativity (Figure 1, top heat map, red area). Additionally, individual typologies accelerated the aging of women (enterprising profession, living in a capital, left-handed, realistic, picnic, compromising) with some exclusions for a few studied resources (top heat map, red columns).

At the same time, in women, to a greater extent than in men, most personal resources can significantly slow down aging (Figure 1, see blue rows). The following resources were negatively correlated with the RBA index (slowing down aging): sport, optimism, communication, nature, achievements, handwork, and kindness. Moreover, this was visible for most individual typologies. The resources intellect, achievements, and order (Figure 1 top heat map) also slowed down aging in some individual typologies.

For men, most personal resources accelerated aging. Resources risk, handwork, kindness, nature, and humor were especially strongly positively correlated with the RBA index (Figure 1, bottom heat map, red rows). However, the men’s heat map showed some individual typologies which were negatively correlated with the RBA index (slow down aging). These include intellectual occupation, left-handed or ambidexterity (Figure 1, bottom heat map, bright blue columns). Cluster analysis also indicated that being left-handed is beneficial for men but disadvantageous for women.

The picture shows clustered correlation coefficients grouped both by personal resources (rows) and by individual typology (columns). The heat map is made to visualize and find related clusters for the data presented in the Supplementary Tables S1–S16. The X-axis represents the different clusters of individuals pre-grouped based on their personal typologies. The scale bar shows the correlation coefficient. The red gradient shows a positive correlation RBA index with personal resources. The blue shows a negative correlation. The scales on the top and to the left indicate the steps of clustering by the Pearson’s correlation.

The negative impact of additional resources on the rate of aging for men, perhaps can be explained by the greater concentration of men in a career, earning material resources and heavy workload, and the enthusiasm for work against the background of a decrease in vitality at this age. While women at this age in Russia concentrate more on family and personal development, leaving their career ambitions in the past.

### 3.2. Stepwise Regression Analysis of the Impact of Personal Resources on the RBA Index

To identify the main personal resources that affect the RBA index, a multiple regression analysis was carried out with a stepwise exclusion of non-essential variables. The dependent variable was the RBA index, and personal resources were used as the independent variables. Regression models were calculated for the entire data set, regardless of individual typologies, but separately for men and women. The results of calculating the coefficients for linear regression analysis are presented in Table 1 for women and in Table 2 for men.

The multiple linear regression model explained 24% of the variance (R2 = 0.245) and the model was significant (F = 74.782 *p* < 0.00000). As can be seen from Table 1, all significant resources (creativity, handwork and optimism) are favorable for women (have negative coefficients). This result was mostly consistent with correlation and cluster analysis (Figure 1 and Supplementary Data), but not for all typologies.

The resulting multiple regression model explained 49% (R2 = 0.493), and the model was significant (F = 72.68 *p* < 0.00000). The coefficients included in the model showed that for men of this age, favorable resources are sports, kindness, optimism, and unfavorable activities are associated with risk and spending time in nature (Table 2). For men, regression analysis revealed a range of unfavorable hobbies. When comparing the results of regression analysis with cluster analysis, it turned out that the benefit or harm of the resource varied greatly depending on the personal typology. To take into account this heterogeneity and increase the accuracy of the model, we applied a more complex analysis, in which the model included both personal resources and individual typologies.

### 3.3. Development of a Simulation Model Using ANN

**Figure 2:**
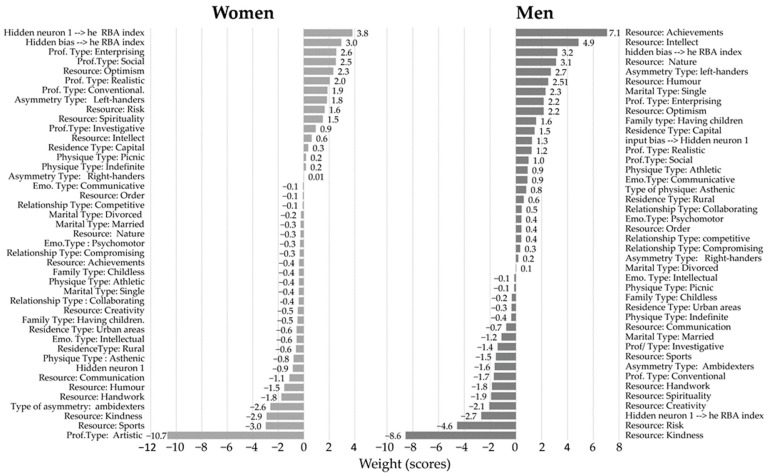
The contribution of personal resources and individual typology to the trained ANN model of biological aging (weight values are shown). Positive values contribute to the acceleration of aging, while negative values contribute to the delay of aging.

For further generalizing analysis and building of a simulation model, we used the automatic artificial neural network (ANN) trained in this study (Figure 2, for explanations, see Section 2.4). Using ANN, we assessed the simultaneous impact of all personal resources and all individual types on the index of biological aging of an individual.

ANN generated a number of personality values that slow down aging (reduce the RBA index) and accelerate aging (the RBA index elevation). Note, that the negative values of weight in Figure 2 report a slowdown in aging (favorable indicators), while positive value weight shows an acceleration in aging (unfavorable indicators).

Note that the ANN strategy does not provide for the calculation of significance levels for the weight of an individual indicator value. To assess the reliability of the results of the trained ANN, the correlation coefficients between the actual values of the biological aging index and the output values obtained using the training and test ANNs were calculated.

Figure 2 shows that the ANNs trained in this study gave a reliable (*p* < 0.001, Table 3) prediction of biological aging based on input indicators of the use of individual resources and individual typological characteristics of the respondent. Note that the simulation model generated by the trained ANN mostly showed results close to those calculated using cluster analysis. Thus, in both analyses in women, the most favorable resources are: sports, kindness, humor, handwork, and communication, and the most favorable personal characteristics: professional type: artistic, type of right-left-sided asymmetry: ambidexters, type of physique: asthenic, type of place of residence: rural, and type of emotionality: intellectual. According to the results from ANN in men, the most favorable personality resources are: kindness, risk, creativity, spirituality, and handwork, and the most favorable personality types are: professional type: conventional and investigative, type of right-left-sided asymmetry: ambidexters, marital status type: married, type of physique: indefinite. However, for men, discrepancies were found between the ANN results and the data of correlation and cluster analysis. For example, in the assessment of the resources kindness and risk. ANN predicted that the contribution of both resources to slowing down aging was favorable (weight: −8.56 and −4.56), (Figure 2). At the same time, according to the analysis of correlations, kindness was included in the cluster of factors accelerating aging for most types of men (except ambidexters).

The impact of risk was more heterogeneous and highly dependent on the man’s personality type (Figure 1, men). Thus, for men, we assumed that personal resources affect different individual types in different ways. Therefore, when selecting personal anti-aging resources, the benefits of kindness and risk should be considered separately depending on the typology of the respondent’s personality.

The trained ANN was used to model the impact of personal resources on aging. For 100 randomly selected subjects, we simulated how the aging index would change if the respondents changed the choice of their personal resources (Table 4, for methods see Section 2.4).

According to the ANN forecast, group optimization of personal resources is quite effective for women. An increase in favorable resources by 1 point for women reduced the biological aging index by 3.4 years compared to empirical values. Whereas for men only individual-typological selection of personal resources is effective. So, only individual selection reduced the index of biological aging by 1.9 years compared with empirical values. Simulation of other scenarios dramatically increased the predicted biological aging index (Table 4).

## 4. Discussion

### 4.1. Personal Resources and Biological Aging

We have proposed our own interpretation of the concept of personal resources. We propose to consider personal resources as compensatory formations, additional to daily life (family and work). Above, we defined personal resources as features of the psyche or behaviors that contribute to the slowdown of biological aging. We assumed that personal resources affect biological aging depending on the individual typological characteristics, and our hypothesis was confirmed. The most important individual typological features was found to be gender. This is consistent with data previously obtained by us [54,58] and by other researchers [59]. The present study shows that, both in men and women, different resources are associated with the relative biological aging index.

First, we will discuss the results obtained for women. We allocated 28 types of respondents based on six characteristics. Unconditionally positive personal resources have negative correlations with the RBA index for many groups of subjects and do not have positive correlations (See Supplementary Tables S1–S16 and Figure 1).

The most effective resources are kindness and communication. They significantly slowed down biological aging in 16 types of women, and did not accelerate it in any type. We assessed resources as the types of activities that the respondent was engaged in during the last year. By kindness, we understood caring for someone (children, parents, pets) or doing charity work. communication was identified as activities associated with other people: visiting or receiving guests, participating in group activities, meeting friends, etc.

Sports. The presence of sports hobbies helped to slow down biological aging in nine types of women (no one has acceleration). By sports, we assumed the presence of sports hobbies (exercising, attending sports groups, hiking, walking, etc.).

Humor slowed down aging in eight types and did not accelerate in any. By humor, we understood interest in anecdotes or jokes, visiting or watching funny shows, etc.

Subject resource. Passion for handwork helped to slow down biological aging in six types of women (no one has an acceleration). We understand the subject resource as the presence of occupations related to working with hands; for women, these are various options of craft, needlework, etc.

Achievements. Achievements and victories in some areas of life were positive for four types of women, it slowed down their biological aging (no one had acceleration). We consider achievements not only as professional accomplishments, although they certainly contribute, but any success: win a competition, take first place, be a winner in some activity. It is important to achieve such success in additional types of activity, in a hobby, that will bring positive emotions which in turn will help slow down aging.

The other six resources were conditionally positive: nature, optimism, order, creativity, intellect, spirituality. We defined the resource nature as interaction with animals or plants (walks in the forest, having plants and animals at home, working in the garden, etc.). Nature was a positive resource (slow down biological aging) for seven types of women and negative (accelerating biological aging) for only one. Optimism is, first of all, a good attitude towards oneself. An optimistic person evaluates themselves, own abilities and prospects as higher than those of others. This quality is not a hobby, but it can be improved through psychological training without fundamentally changing the entire life path. Optimism was a positive resource for seven types of women and a negative resource for two. By creativity, we mean the presence of hobbies associated with both productive (composing poems, stories, music, etc.) and reproductive creativity (reading books, visiting art salons, exhibitions, theatre, etc.). creativity was a positive resource for three types of women and a negative resource for two. We defined intellect as the presence of intellectual hobbies, enthusiasm for the news of science and technology, education and self-education. Intellect was a positive resource for three types of women and a negative-for two types. The resource order is effective time management: drawing up preliminary plans, observing the daily routine, controlling expenses, etc. Order was a positive resource for two types of women and a negative resource for one. By spirituality, we understood all types of practices related to self-improvement, interest in the existential-religious field. Spirituality was a positive resource for four types of women and a negative resource for seven.

The resource risk is all kinds of risky hobbies and activities. It appeared to be negative for all women, it accelerated biological aging in fourteen groups of women and did not slow it down in anyone. However, we still consider it as the personal resource, since it is one of the few resources that is positive for one type of men (slow down biological aging). In the male sample, the results were quite different. Moreover, resources that were positive for women were negative for men and vice versa.

In the first place for men were the resources of sports and creativity. Both resources slowed down biological aging in one type, but did not accelerate it in anyone. These are certainly positive resources that can be recommended to men, as they will not harm. Additionally, all other resources need to be selected considering individuality only. In second place, we put resources that were positive for at least one type, but negative for other types: intellect, communication, optimism, spirituality, and risk. Intellect slowed down aging in one type, and accelerated it in one. Communication slowed down aging in one type, and sped up in two. Optimism slowed down aging in two types, it also accelerated aging in two. Spirituality slowed down aging in one type, and sped up in four. Risk slowed aging in one type, and accelerated in thirteen.

These observations suggest that it is necessary to select personal resources considering the person’s individuality. It is important to consider what type a person belongs to according to all selected parameters of individuality (gender, age, place of residence, marital status, professional type, body type, functional asymmetry, emotionality, and style of relationships with other people) and, based on that calculate the total indicator of the advantageousness of each resource. Individual selection of personal resources is a further perspective of our research.

Notably, many resources for men of pre-retirement age turned out to be ineffective (achievements, order, humor, nature, handwork, and kindness). The presence in individual life of such activities (in the form of hobbies and interests) results in an acceleration in biological aging, rather than slowdown. We believe that this paradox result is related to our interpretation of the concept of personal resources and the age of the respondents. We consider personal resources as activities that are additional to daily life. In other words, these are hobbies and activities that help a working adult compensate for the load and maintain relative youth. However, the age we chose for the study, 35–60 years, is the period of building a professional career. The main working activity falls during this period. Before, a person can search the career path, try different options. After this period (60–65), they retire. For many men, professional success is more important than for women; they put more effort into work, do not want to be distracted, and perceive failure more dramatically. This explanation is supported by other researchers’ studies. For example, in Austria, men who retire a year early are more likely to die than those who continue to work (by 13%). Additionally, with forced firing, the risk of premature death for men increases significantly. However, among women, mortality remains practically unchanged, which is explained by the involvement of women in the household and family affairs, while men who lose their jobs also lose their social status as a family provider (breadwinner) without receiving another role in return [60].

We believe that men of the studied age group are very much focused on professional activities, for them either the activity itself or family life acts as personal resources. For men, additional activities rarely become a source of anti-aging, they distract them from their main activities and are perceived negatively. Unlike them, most women of this age group need additional activities to slow down biological aging. This proposed explanation needs further investigation.

In the male sample, there were significantly fewer resources that were negatively correlated with the biological aging index, and more positively correlated. Above, we explained this by the fact that men at this age in Russia are more occupied with a career, and their main favorable resource is professional activity (career, wages, the role of the main breadwinner of the family, or mortgage payer), additional activities in the form of hobbies or grouping distract from their career and act as an additional load.

The results of the multivariate linear regression analysis confirmed our findings obtained from the cluster analysis. Regression analysis for men and women samples without dividing them into individual typologies revealed in women that all significant resources have a negative correlation with the biological aging index, which means they are favorable. These are creativity, optimism, and handwork. “Bad” resources that give a positive contribution to the RBA index were not found. This means that all personal resources for women are either favorable, conditionally favorable or neutral (Table 1). According to the values of the regression coefficients for men, along with favorable resources (negative coefficients), unfavorable resources (positive coefficients) were found. Favorable resources included sports, kindness, and optimism. Unfavorable resources were risk, nature, and creativity (Table 2). Most likely, these are precisely the types of activities that are either dangerous to health (risk) or serve as an explanation for professional unfulfillment. For example, men’s involvement in creative hobbies may be a defensive reaction, compensation for career failures or routine working conditions. For men, the nature resource most often appears in the form of fishing trips, barbecues, which can be accompanied by alcohol consumption and other bad habits. All these assumptions require additional study and statistical evaluation.

However, it should be noted that such general patterns explain only a small part of the variance in the sample (24% for women and 49% for men). We believe that the rest of the variance is explained by typological or individual characteristics. To understand this, we conducted further analysis using automatic neural networks (ANNs).

### 4.2. Analysis of Theoretical Models Generated by Artificial Neural Network

For women, the ANN trained in the study predicted the ability to use favorable personal resources to slow biological aging. The simulation showed that if women make more use of personal resources that are favorable for them (sports, kindness, or handwork), then their biological aging index will decrease by an average 3.4 years compared to empirical values. They will begin to feel younger and health indicators will also improve. The same resources negatively correlate with the biological aging index for women. This was also confirmed both by correlation analysis separately for individual personality typologies and by cluster analysis.

However, the simulation for men gave a more complex picture. The trained ANN showed that the selection of personal resources for all men in the sample is not effective in reducing the index of biological aging. If we increased the level of use of favorable resources by one point in the men sample (kindness, risk, or creativity), then the indicator of biological aging increased by 52 years. If we reduced the level of use of unfavorable resources by one point (nature, intelligence, or achievements), then the aging indicator still increased by 8 years. We assumed that there are no universal personal resources favorable for all men. Therefore, each man needs to select resources individually, taking into account all his typological features. To confirm this notion, we simulated individual selection by changing the use of favorable and unfavorable resources by one point and taking into analysis a sample of men with an increased index of biological aging. To select resources, we were guided by the identified patterns of correlation (see Supplementary Tables S9–S16 and Figure 1). Using ANN for modeling, we decreased for each respondent in the sample the resources positively correlated with aging by one point, and increased by one point resources with a negative correlation (given the typology of a man each time). As a result of simulation, the predicted biological aging index decreased by 1.9 years (Table 4). Thus, for men, one can also choose individual resources that slow down their aging. However, to solve the problem of slowing down aging in men, it is required to take into account individual typologies when choosing resources that affect aging.

It should be noted once again that all the situations analyzed in this section are not obtained empirical data. These are theoretical model simulations based on our ANN trained on empirical data. Here, we wanted to emphasize the prospects for using trained neural networks for group or individual selection of anti-aging personal resources. Indeed, in order to test the effectiveness of our predictions, additional active in-field experiments are needed to study the real changes in the rate of aging depending on the applied resources. In the future, ANN training, for the selection of personal resources to compensate for the negative effects of social changes and upheavals in life, may contribute to improving the health and active longevity of the population.

### 4.3. Evaluation of Data and Methods Limitations

We developed methods for assessing personal resources in accordance with our concept and the criteria described above. Conclusions and recommendations were made corresponding to our proposed approach. Personal resources are activities that are additional to the respondent’s daily life, which are amenable to training and development (mainly a variety of hobbies and interests). For example, by intellect, we understood the presence of intellectual hobbies, and not an IQ score. The findings cannot be extended to other understandings of personal resources without additional research. An empirical study was performed on the pre-retirement age group (men 36–60 years old, women 36–55 years old). The outcomes cannot be generalized to other age groups; moreover, we believe that other age groups will have different results. The work on neural network training is a pilot study and requires further development of the model on a larger sample with additional personal resources and typologies. In the current state, the automatic neural network (ANN) we trained is more suitable for predicting the rate of aging in women than in men.

Further research will be continued on larger samples and using additional typologies, taking into account the identified limitations of the assessment of personal resources for more accurate and personalized forecasting of the RBA index using ANNs.

### 4.4. Research Perspectives

We see the perspectives of our work in performing the study of biological age resources in other age groups. It is necessary to check whether our findings regarding the male sample are valid for other ages. We assume that the results obtained are related to the relevant stage of the life path (work and career growth) and should not be retained for other age periods. We would also like to carry out a practical implementation of the results obtained, namely, based on the patterns gained, develop an ANN-based computer program considering respondents’ typology for selecting personal resources for specific anti-aging strategies.

## 5. Conclusions

We proposed a novel approach to defining personal anti-aging resources as complementary activities that a person can master and use subjectively, in addition to their daily life (family and professional). This activity can improve health, decelerate biological aging, and feasibly increase life expectancy. In this study, 13 resources were explored including sports, order, creativity, intellect, handwork, kindness, humor, spirituality, risk, communication, nature, achievements, and optimism. It was revealed that the effectiveness of the allocated personal resources depends on the individual typology of a person, i.e., age group, profession, marital status, place of residence, etc.

The effectiveness of personal resources was analyzed separately for women and men and showed different patterns of correlations and interactions between resources and typologies. In the women sample, 92% of the resources contributed to slowing down biological aging in at least one type of respondent. In men, only 54% of the personal re-sources contributed to a decrease in the RBA index for at least one type, and 44% of the resources increased the RBA index, i.e., they acted as negative factors.

Cluster analysis of correlations also showed that for women, personal resources have a great impact on slowing down aging. Almost all individual resources, especially sport, optimism, handwork, and kindness, regardless of typology, remain significant anti-aging factors. In men, individual typologies have a greater impact on slowing down aging than personal resources. For example, the intellectual type of occupation or functional asymmetry (ambidexterity or left-handedness) negatively correlated with the biological aging index for most personal resources in the study (Figure 1).

Using the ANNs trained in this study, we selected personal resources and typologies that slow down aging. For women, group selection of resources is feasible. If all women will use more often resources nature, intellect, and achievements, then their biological aging will slow down. For men, group selection of resources is difficult. However, the ANN trained in this study allocates favorable and unfavorable resources for each man individually, taking into account the peculiarities of their personal typology.

## Figures and Tables

**Table 1:** Multiple linear regression equation coefficients for women.

Variables	Coefficients	*p* Values
Constant value	5.1048	0.000000
Creativity	−1.2047	0.000290
Subject resource (Handwork)	−1.5232	0.000000
Optimism	−1.2957	0.000000

**Table 2:** Multiple linear regression equation coefficients for men.

Variables	Coefficients	*p* Values
Constant value	7.2429	0.000000
Sports	−2.8716	0.000000
Creativity	1.5160	0.000013
Kindness	−1.4834	0.000020
Risk	1.0961	0.000103
Nature	1.8193	0.000000
Optimism	−2.4004	0.000000

**Table 3:** Correlation coefficients between actual biological aging index and output values generated by training and test ANNs.

	Training Neural Network	Testnet (Minimum)	Testnet (Maximum)
Men	0.684967 ***	0.561021 ***	0.693855 ***
Women	0.700902 ***	0.629518 ***	0.629522 ***

*** *p* < 0.001.

**Table 4:** Trained ANN forecast for the aging index of men and women when the personal resources of the respondents varied.

	Empirical Aging Index (Relative Years)	Predicted Aging Index after 1 Point Increase in Favorable Resources. (Relative Years)	Predicted Aging Index after a 1 Point Decrease in Adverse Resources. (Relative Years)	Predicted Aging Index after an Individual Decrease in Adverse Resources by 1 Point and an Increase in Favorable Resources by 1 Point (According to Personality Typology). (Relative Years)
Women	−0.4 ± 9.90	−3.8 ± 0.78	Not done	Not done
Men	2.5 ± 4.28	55.4 ± 88.04	10.4 ± 42.44	0.6 ± 0.01

## Data Availability

Data available on request due to ethical restrictions, and in accordance with the Federal Law “On the Protection of Personal Data” (The Russian Federation of 27 July 2006 N 152-FZ).

## Supplementary Materials

The following supporting information can be downloaded at: https://www.mdpi.com/article/10.3390/ejihpe12120126/s1, Supplementary Tables S1–S16: Correlation between the RBA index and personal resources.
