# Supplementary material for: Use of Personal Resources May Influence the Rate of Biological Aging Depending on Individual Typology
Source: Eur J Investig Health Psychol Educ. 2022 Dec 2;12(12):1793–811. doi: 10.3390/ejihpe12120126 (PMC9778189; doi:10.3390/ejihpe12120126)
Supplement: Supplementary file 1 [file ejihpe-12-00126-s001.zip › ejihpe-1898170-supplementary.pdf]

Supplementary Table S1. Correlation between the RBA index and personal resources among representatives of different professional types (women).

**Types**	***Sports***	***Order***	***Creativity***	***Intellect***	***Handwork***	***Kindness***	***Humour***	***Spirituality***	***Risk***	***Communication***	***Nature***	***Achievements***	***Optimism***
**1**	**0.01**	**0.12**	**0.18**	**-0.03**	**-0.15**	**-0.42 *****	**0.13**	**0.02**	**0.09**	**-0.28***	**0**	**-0.09**	**-0.27***
**2**	**-0.28**	**-0.48 ****	**-0.3**	**-0.33***	**-0.03**	**0.03**	**-0.43 ****	**-0.47 ****	**0.16**	**-0.4***	**-0.12**	**0.02**	**-0.47 ****
**3**	**-0.1**	**0.13**	**0**	**0.1**	**0**	**-0.21 ****	**-0.15***	**0.04**	**0.16***	**-0.23 *****	**0.01**	**-0.06**	**-0.01**
**4**	**-0.09**	**-0.36***	**0.31***	**-0.22**	**-0.26**	**-0.37***	**-0.08**	**0.42* ***	**0.48* ***	**-0.27**	**-0.47 ****	**-0.32***	**-0.27**
**5**	**-0.11**	**-0.01**	**0.18**	**0.41**	**-0.85 *****	**0.37**	**0.11**	**-0.02**	**-0.14**	**-0.07**	**-0.23**	**0.29**	**0.5***

1 - Realistic, 2 - Investigative, 3 - Social, 4 - Conventional, 5 - Enterprising type, (* - p<0.05; ** - p<0.01; *** - p<0.001)

In the Supplementary Table 1, among the female representatives of professions of the *Realistic* type, the RBA index is negatively associated with the resources *Kindness, Communication, Optimism*. For the female representatives of *Investigative* professions, the RBA index is negatively associated with resources *Order, Intellect (intellectual hobbies), Humour, Spirituality, Communication, Optimism*. In female representatives of the *Social* type of professions, the RBA index is negatively related to the following resources: *Kindness, Humour, Communication*, and positively - to the resource *Risk* (the presence of risky hobbies). In women representatives of the *Conventional* type of professions, the RBA index is negatively related to resources *Order, Kindness, Nature, Achievements*; and positively - to *Creativity, Spirituality, Risk*. The female representatives of the *Enterprising* type of professions have an RBA index that negatively correlates with the *Handwork* resource (subject hobbies), and positively - with the resource *Optimism*.

Supplementary Table S2. Correlation between the biological aging index and personal resources in representatives of different family types (women).

Types	***Sports***	***Order***	***Creativity***	***Intellect***	***Handwork***	***Kindness***	***Humour***	***Spirituality***	***Risk***	***Communicatio n***	***Nature***	***Achievements***	***Optimism***
1	-0.2	-0.09	0.08	-0.18	-0.14	-0.29 **	0.14	0.43 ***	0.47 ***	-0.24*	-0.16	-0.29 **	-0.22*
2	-0.02	-0.03	0.17*	-0.01	0.05	-0.27 **	-0.15	0.12	0.1	-0.43 ***	-0.11	-0.14	-0.22*
3	-0.24 **	0.06	-0.11	0.07	-0.14	-0.11	-0.17*	-0.18 *	0.1	-0.1	-0.07	-0.01	0.02

1 - singles, 2 – divorced, 3 – married, (* - p<0.05; ** - p<0.01; *** - p<0.001)

As the Supplementary Table 2 shows, in single women, the RBA index is negatively related to resources *Kindness, Communication, Achievements, Optimism*, and positively - to Spirituality and *Risk*. In divorced women, the biological aging index is negatively associated with resources *Kindness,*

Communication, Optimism, and positively - with *Creativity*. In married women, the RBA index negatively correlated to resources *Sports, Humour, Spirituality*.

Supplementary Table S3. Correlation between the biological aging index and personal resources in women with and without children.

Types	***Sports***	***Order***	***Creativity***	***Intellect***	***Handwork***	***Kindness***	***Humour***	***Spirituality***	***Risk***	***Communication***	***Nature***	***Achievements***	***Optimism***
1	-0.06	0.02	0.11	-0.07	0.01	-0.27*	-0.02	0.42**	0.6 ***	-0.18	-0.11	-0.08	-0.09
2	-0.14*	0	0.03	-0.01	-0.13*	-0.22 ***	-0.1	-0.02	0.12*	-0.25 ***	-0.11*	-0.12*	-0.1

1 - childless, 2 - having children, (* - p<0.05; ** - p<0.01; *** - p<0.001)

Supplementary Table 3 shows that in women who do not have children, the biological aging index is negatively related to resource *Kindness*, and positively - to *Spirituality* and *Risk*. In women with children, the RBA index is negatively associated with resources *Handwork, Kindness, Nature, Communication, Achievements*, and positively - with *Risk*.

Supplementary Table S4. Correlation between the RBA index and personal resources in women with different types of functional asymmetry.

Types	***Sports***	***Order***	***Creativity***	***Intellect***	***Handwork***	***Kindness***	***Humour***	***Spirituality***	***Risk***	***Communication***	***Nature***	***Achievements***	***Optimism***
1	0.21	0.46	0.39	0.35	-0.47	-0.66*	0.48	0.8**	0.68*	0.33	-0.15	0.49	0.61*
2	0.46	-0.29	0.37	-0.08	0.11	-0.2	0.19	-0.36	0.65 **	-0.79 ***	-0.37	-0.1	-0.38
3	-0.18 **	0	-0.01	-0.01	-0.09	-0.2**	-0.13*	-0.01	0.15*	-0.22 ***	-0.1	-0.11	-0.1

1 - left-handed, 2 – ambidexter, 3 - right-handed, (* - p<0.05; ** - p<0.01; *** - p<0.001)

As can be seen in the Supplementary Table 4, in left-handed women, the RBA index is negatively associated with the resources Kindness and positively - with the resources *Spirituality, Risk, Optimism*. In ambidexter women, the RBA index is negatively related to the *Communication* resource and positively - to the *Risk* resource. In right-handed women, the RBA index negatively correlates with resources *Sports, Kindness, Humour, Communication*, and positively - with *Risk*.

Supplementary Table S5. Correlation between the RBA index and personal resources in representatives of different body types (women).

Types	***Sports***	***Order***	***Creativity***	***Intellect***	***Handwork***	***Kindness***	***Humour***	***Spirituality***	***Risk***	***Communication***	***Nature***	***Achievements***	***Optimism***
1	-0.06	-0.3*	-0.17	-0.02	0.12	-0.05	-0.35*	-0.25	0.06	-0.56 ***	-0.17	-0.22	-0.22

2	-0.04	0.14	0.08	0.09	-0.28 ***	-0.35 ***	-0.01	0.01	0.06	-0.37 ***	0.18 *	0.01	-0.18*
3	-0.15	-0.1	0.17	-0.4	0.21	-0.02	0.09	-0.61 ***	0.1	-0.44 **	-0.42 **	-0.24	-0.25
4	-0.17*	0.07	0.01	0.01	-0.12	-0.23 **	-0.08	0.3 ***	0.29 ***	0	-0.2*	-0.14	0.07

1 - asthenic, 2 - picnic, 3 - athletic, 4 - indefinite (harmonious), (* - p<0.05; ** - p<0.01; *** - p<0.001).

Supplementary Table 5 demonstrates that, in asthenic women, the RBA index is negatively related to resources *Order, Humour, Communication*. In picnic women, the RBA index is negatively associated with resources *Handwork, Kindness, Communication, Optimism*. In female athletes, the RBA index negatively correlates with resources *Spirituality, Communication, Nature*. In women of indefinite physique, the RBA index is negatively related to resources *Sports, Kindness, Nature*, and positively - to resources *Spirituality* and *Risk*.

Supplementary Table S6. Correlation between the RBA index and personal resources in representatives of different types of emotionality (women).

Types	***Sports***	***Order***	***Creativity***	***Intellect***	***Handwork***	***Kindness***	***Humour***	***Spirituality***	***Risk***	***Communication***	***Nature***	***Achievements***	***Optimism***
1	-0.31 **	-0.09	-0.23 *	-0.11	-0.26*	-0.27 **	-0.19	-0.37 ***	0	-0.47 ***	-0.11	-0.11	-0.48 ***
2	-0.02	-0.12	-0.14	0.09	0.09	-0.11	-0.22	-0.02	0.27*	-0.31 **	-0.32 **	-0.15	-0.08
3	-0.13	0.05	0.13	0	-0.08	-0.17 *	-0.07	0.16*	0.19*	-0.1	-0.08	-0.11	0

1 - psychomotor, 2 - intellectual, 3 - communicative, (* - p<0.05; ** - p<0.01; *** - p<0.001).

In women with psychomotor emotionality, the RBA index is negatively related to resources *Sports, Creativity, Handwork, Kindness, Spirituality, Communication, Optimism*. In women with intellectual emotionality, the RBA index negatively correlates with resources *Communication, Nature*, and positively - with *Risk*. In women with communicative emotionality, the RBA index is negatively associated with resource *Kindness* and positively - with *Spirituality* and *Risk*.

Supplementary Table S7. Correlation between the RBA index and personal resources among representatives of different types of interaction (women).

Types	***Sports***	***Order***	***Creativity***	***Intellect***	***Handwork***	***Kindness***	***Humour***	***Spirituality***	***Risk***	***Communication***	***Nature***	***Achievements***	***Optimism***
1	-0.52 **	-0.35	-0.14	-0.61 ***	-0.33	-0.01	-0.26	-0.04	0.42*	-0.17	-0.12	-0.28	-0.29
2	0.05	0.2*	0.26 **	0.25**	-0.18*	-0.31 ***	0.01	0.18*	0.23 **	-0.19*	0.04	0.13	-0.04
3	-0.25 **	-0.11	-0.27 ***	-0.12	0.09	-0.12	-0.19*	-0.12	0.01	-0.33 ***	-0.3 ***	-0.3 ***	-0.1

1 - competitive, 2 - compromising, 3 - collaborating, (* - p<0.05; ** - p<0.01; *** - p<0.001).

As can be seen in the Supplementary Table 7, in women who prefer competition in relationships, the RBA index is negatively related to resources *Sports, Intellect*, and positively - to *Risk*. In women who choose a compromise, the RBA index negatively correlates with resources *Handwork, Kindness,*

Communication and positively - with resources *Order, Creativity, Intellect, Spirituality, Risk*. In women who prefer cooperation in interactions, the RBA index is negatively associated with resources *Sports, Creativity, Humour, Communication, Nature, Achievements*.

Supplementary Table S8. Correlation of the RBA index with personal resources for women living in different types of residence area.

Types	*Sports*	*Order*	*Creativity*	*Intellect*	*Handwork*	*Kindness*	*Humour*	*Spirituality*	*Risk*	*Communication*	*Nature*	*Achievements*	*Optimism*
1	-0.2*	0.08	-0.14	0.03	0.07	-0.14	0.11	-0.18	-0.05	-0.08	-0.12	-0.05	-0.04
2	-0.17*	-0.15*	-0.09	-0.24 ***	-0.06	-0.27 ***	-0.16*	-0.04	0.12	-0.4 ***	-0.17*	-0.32 ***	-0.26 ***
3	-0.14	0.09	0.08	0.36*	-0.33*	0.02	-0.31*	0.17	0.33*	0.11	-0.03	0.15	0.19

1 – rural areas, 2 - urban areas, 3 - capital, (* - p<0.05; ** - p<0.01; *** - p<0.001).

For women living in rural areas, the RBA index negatively correlates with resource *Sports*. For women living in urban areas, the RBA index is negatively related to resources *Sports, Order, Intellect, Kindness, Humour, Communication, Nature, Achievements, Optimism*. For women living in the capital, the RBA index is negatively associated with resources *Intellect, Handwork, Humour*, and positively - with resources *Intellect* and *Risk*. Results of correlation analysis for the male sample are presented in Supplementary Tables 9-16.

Supplementary Table S9. Correlation between the RBA index and personal resources among representatives of different professional types (men).

Types	*Sports*	*Order*	*Creativity*	*Intellect*	*Handwork*	*Kindness*	*Humour*	*Spirituality*	*Risk*	*Communication*	*Nature*	*Achievements*	*Optimism*
1	-0.03	-0.03	-0.12	0.01	0.21 **	0.29 ***	0.14	0.05	0.19 *	0.08	0.2*	0.09	0.17*
3	0.28	0.12	0.16	-0.01	0.22	0.24	0.18	0.12	0.23	-0.06	0.23	-0.1	-0.14
4	-0.1	-0.45	0.42	-0.09	0.11	0.34	0.01	0.57*	- 0.44	-0.51	0.02	0.76 **	-0.22
5	-0.09	-0.05	-0.26	0.17	0.21	-0.11	0.3	-0.55 **	- 0.53 **	0.4*	0.03	0.13	-0.09

1 - Realistic, 2 – Investigative (insignificant respondent amount in the sample, for explanation see material and method chapter), 3 - Social, 4 - Conventional, 5 - Enterprising type, (* - p<0.05; ** - p<0.01; *** - p<0.001).

As can be seen in the Supplementary Table 9, in male representatives of the *Realistic* type of professions, the RBA index is positively associated with resources *Handwork, Kindness, Risk, Nature, Optimism*. In men - representatives of the *Social* type of professions, no significant correlations were found. In male representatives of the *Conventional* type of professions, the RBA index is positively associated with resources *Spirituality* and *Achievements*. In male representatives of the *Enterprising* type of professions, the RBA index is negatively correlated with resources *Spirituality, Risk*, and positively - with the resource *Communication*.

Supplementary Table S10. Correlation between the RBA index and personal resources in representatives of different family types (men).

Types	*Sports*	*Order*	*Creativity*	*Intellect*	*Handwork*	*Kindness*	*Humour*	*Spirituality*	*Risk*	*Communication*	*Nature*	*Achievements*	*Optimism*
1	0.01	0.28*	0.08	0.05	-0.01	0.13	0.28*	0.16	0.37 **	0.21	0.01	0.1	-0.05
2	0.01	0.28*	0.08	0.05	-0.01	0.13	0.28*	0.16	0.37 **	0.21	0.01	0.1	-0.05
3	-0.08	-0.09	0.05	0.06	0.31 ***	0.27 **	0.03	0.08	0.11	-0.06	0.23*	0.01	0.01

1 - singles, 2 – divorced, 3 – married, (* - p<0.05; ** - p<0.01; *** - p<0.001).

As the Supplementary Table 10 shows, in single men, the RBA index is positively associated with resources *Order, Humour, Risk*. The same correlation is observed in divorced men. In married men, the RBA index is positively related to resources *Handwork, Kindness, Nature*.

Supplementary Table S11. Correlation between the biological aging index and personal resources in men with and without children.

Types	*Sports*	*Order*	*Creativity*	*Intellect*	*Handwork*	*Kindness*	*Humour*	*Spirituality*	*Risk*	*Communication*	*Nature*	*Achievements*	*Optimism*
1	0.06	0.32*	0.05	0.04	-0.13	-0.01	0.16	0.11	0.3*	0.1	-0.09	-0.08	-0.14
2	0	-0.09	-0.02	0.01	0.35 ***	0.35 ***	0.17*	0.05	0.18 **	0	0.28 ***	0.08	0.17*

1 - not having children, 2 - with children, (* - p<0.05; ** - p<0.01; *** - p<0.001).

For men without children, the RBA index of biological aging is positively associated with resources *Order* and *Risk*. For men with children, the RBA index positively correlates with resources *Handwork, Kindness, Humour, Risk, Nature, Optimism.*

Supplementary Table S12. Correlation between the RBA index and personal resources in men with different types of functional asymmetry.

Types	*Sports*	*Order*	*Creativity*	*Intellect*	*Handwork*	*Kindness*	*Humour*	*Spirituality*	*Risk*	*Communication*	*Nature*	*Achievements*	*Optimism*
1	-0.5*	-0.29	-0.59 *	0.65**	0.85 ***	0.69 **	0.29	-0.85 ***	0.35	-0.69 **	-0.01	0.18	-0.69 **
2	-0.53	-0.33	0.08	-0.62*	0.44	-0.5	-0.08	-0.09	0.62*	0.57*	-0.33	-0.33	-0.64*
3	0.03	0	0.02	-0.02	0.2**	0.27 ***	0.17*	0.1	0.2**	0.02	0.2**	0.02	0.11

1 - left-handed, 2 – ambidexter, 3 - right-handed, (* - p<0.05; ** - p<0.01; *** - p<0.001).

As can be seen in the Supplementary Table 12, in left-handed men, the RBA index is negatively related to resources *Sports, Creativity, Spirituality, Communication, Optimism*, and positively - to resources *Intellect, Handwork, Kindness*. In ambidexter men, the RBA index is negatively associated with resources

Intellect, Optimism, and positively - with resources *Risk* and *Communication*. In right-handed men, the RBA index positively correlates with resources *Handwork, Kindness, Humour, Risk, Nature*.

Supplementary Table S13. Correlation between the RBA index and personal resources in representatives of different body types (men).

Types	*Sports*	*Order*	*Creativity*	*Intellect*	*Handwork*	*Kindness*	*Humour*	*Spirituality*	*Risk*	*Communication*	*Nature*	*Achievements*	*Optimism*
1	0.1	-0.17	-0.23	-0.02	-0.28	0.32*	0.01	0.13	0.15	-0.08	0	-0.22	0.32*
2	0.29	0.1	0.08	0.22	0.55 ***	0.59 ***	0.5**	0.08	0.48 **	0.29	0.49 **	-0.05	0.24
3	-0.1	0	-0.04	-0.01	0.27 **	0.03	0.08	0.22 **	0.24 **	-0.08	0.03	-0.05	-0.08
4	0.02	-0.01	0.05	0.01	0.23*	0.41 ***	0.28 **	-0.07	0.24*	0.06	0.38 ***	0.15	0.15

1 - asthenic, 2 - picnic, 3 - athletic, 4 - indefinite (harmonious) , (* - p<0.05; ** - p<0.01; *** - p<0.001).

As can be seen in the Supplementary Table 13, in asthenic men the RBA index is positively related to resources *Kindness, Optimism*. In picnic men, the RBA index is positively related to resources *Handwork, Kindness, Humour, Risk, Nature*. In male athletes, the RBA index is positively associated with resources *Handwork, Spirituality, Risk*. In men of indefinite physique, the RBA index is positively associated with resources *Kindness, Humour, Risk, Nature*.

Supplementary Table S14. Correlation between the RBA index and personal resources in representatives of different types of emotionality (men).

Types	*Sports*	*Order*	*Creativity*	*Intellect*	*Handwork*	*Kindness*	*Humour*	*Spirituality*	*Risk*	*Communication*	*Nature*	*Achievements*	*Optimism*
1	-0.14	-0.17	0.13	0.1	0.31**	0.2	-0.04	-0.07	0.09	0.12	0.2	-0.11	0.01
2	-0.21	-0.02	0.13	0.21	-0.26	-0.04	0.02	0.11	0.15	-0.11	-0.32	0	-0.36
3	0.12	0.09	-0.09	-0.15	0.27 ***	0.36 ***	0.3 ***	0.07	0.27 ***	0	0.31 ***	0.12	0.22**

1 - psychomotor, 2 - intellectual, 3 - communicative, (* - p<0.05; ** - p<0.01; *** - p<0.001).

As can be seen in the Supplementary Table 14, in men with psychomotor emotionality, the RBA index is positively associated with resource *Handwork*. No significant correlations were found in men with intellectual emotionality. In men with communicative emotionality, the RBA index is positively associated with resources *Handwork, Kindness, Humour, Risk, Nature, Optimism*.

Supplementary Table S15. Correlation between the RBA index and personal resources among representatives of different types of interaction (men).

Types	*Sports*	*Order*	*Creativity*	*Intellect*	*Handwork*	*Kindness*	*Humour*	*Spirituality*	*Risk*	*Communication*	*Nature*	*Achievements*	*Optimism*

1	0.3	0.02	-0.02	-0.17	0.33*	0.54 ***	-0.05	0.41 **	-0.05	-0.21	0.33*	0.27	0.27
2	-0.08	-0.12	-0.03	0.07	0.16	0.25*	0.18	-0.14	0.13	-0.05	0.22*	-0.19	-0.05
3	0.02	0.14	0.03	0.08	0.2*	0.25 **	0.19*	0.2*	0.35 ***	0.06	0.16	0.26 **	0.13

1 – competitive, 2 - compromising, 3 - collaborating, (* - p<0.05; ** - p<0.01; *** - p<0.001).

As can be seen in the Supplementary Table 15, in men who prefer rivalry in relationships, the RBA index is positively associated with resources *Handwork, Kindness, Spirituality, Nature*. In men who prefer compromise, the RBA index is positively associated with resources *Kindness, Nature*. In collaborating men, the RBA index is positively associated with resources *Handwork, Kindness, Humour, Spirituality, Risk, Achievements*.

Supplementary Table S16. Correlation of the RBA index with personal resources for men living in different types of residence area.

Types	*Sports*	*Order*	*Creativity*	*Intellect*	*Handwork*	*Kindness*	*Humour*	*Spirituality*	*Risk*	*Communication*	*Nature*	*Achievements*	*Optimism*
1	-0.14	-0.18	-0.01	-0.11	0.27**	0.29 **	0.01	0.09	0.14	-0.03	0.28 **	0.01	0.13
2	0.1	0.03	-0.18	0.01	0.19	0.2*	0.28 **	0.03	0.28 **	0.07	0.15	-0.1	0.02
3	0.13	0.02	0.16	0.11	0.05	0.26	0.21	0.07	0.17	-0.02	0.08	0.09	-0.04

1 – rural areas, 2 - urban areas, 3 - capital, (* - p<0.05; ** - p<0.01; *** - p<0.001).

As can be seen the Supplementary Table 16, for men living in rural areas, the RBA index is positively associated with resources *Handwork, Kindness, Nature*. For men living in urban areas, the RBA index is positively associated with resources *Kindness, Humour, Risk*. No significant correlations were found among men living in the capital.
